# “Meaninglessness makes me unhappy”: examining the role of a sense of alienation and life satisfaction in the relationship between the presence of meaning and depression among Chinese high school seniors

**DOI:** 10.3389/fpsyt.2025.1494074

**Published:** 2025-02-06

**Authors:** Xiaoxu Hou, Jinsheng Hu, Zhihong Liu

**Affiliations:** College of Psychology, Liaoning Normal University, Dalian, China

**Keywords:** presence of meaning, life satisfaction, sense of alienation, depression, high school senior

## Abstract

**Introduction:**

Given the high incidence of depression among adolescents and its serious consequences, investigating its influencing factors and mechanisms is of great theoretical and practical significance. This study aims to explore the mediating effects of a sense of alienation and life satisfaction on the relationship between the presence of meaning in life and depression among Chinese high school seniors.

**Methods:**

Six hundred and twenty-one senior high school students (17.09 ± 0.45 years, 266 boys) were recruited from Shandong, China, to participate in the study. Participants completed the Adolescent Students’ Sense of Alienation (ASAS), Meaning in Life Questionnaire (MLQ), Satisfaction with Life Scale (SWLS), and Beck Depression Inventory-II (BDI-II).

**Results:**

Our findings revealed that (1) the sense of alienation and life satisfaction play a chain mediating role between the presence of meaning in life and depression among Chinese high school seniors; (2) the sense of alienation plays a mediating role between the presence of meaning in life and depression; (3) there are gender differences in the chain mediation model of the influence of the presence of meaning in life on depression.

**Conclusion:**

This study reveals potential pathways through which the presence of meaning in life affects depression among Chinese high school seniors, offering support and a basis for future mental health interventions for this population.

## 1 Introduction

Adolescence is an important period for psychological and physiological development as well as a high-risk period for depression (1). A recent survey showed that the incidence of depression among 15–20-year-olds in China is far higher than that in other age groups (2). Depression can be damaging to the development of adolescents by impairing cognitive function (3), leading to interpersonal problems and even suicide (4, 5). Given the high incidence of depression among adolescents and its serious consequences, exploring its influencing factors and mechanisms is of great theoretical and practical significance.

Erikson (6) pointed out that adolescents experience a period of role and identity confusion. Finding and establishing meaning in life is a critical issue for adolescents to establish their self-identity. It is also an important task that they must complete (7), which will have a significant impact on their mental health (8, 9). Meaning in life refers to the choice and pursuit of valuable goals and one’s existence, a sense of coherence in one’s life, and the nature of one’s existence (10–12). Studies on the meaning in life have paid more attention to the elderly and adults (13–15). Although researchers have begun to focus on adolescents’ meaning in life (16, 17) and have found that it is also an important factor in a series of mental health problems among adolescents (18, 19), research is still insufficient. Considering the significance of meaning in life in the development of adolescent mental health and the high incidence of depression, this study further explored the relationship between meaning in life and depression in adolescents and its underlying mechanism.

Meaning in life can help individuals better understand their existence, inspire them to pursue meaningful ideals, and achieve their life goals (20, 21). Individuals with a strong sense of meaning in life often prioritize living in alignment with their values and goals. They also possess the courage to engage in self-transcending activities, which enhances self-satisfaction and promotes better mental health and emotional well-being (22). Brassai et al. (23) found that meaning in life not only serves as a crucial protective factor for adolescents’ mental health, but also reduces high-risk behaviors, such as alcohol abuse and drug use, that can harm their physical health. Avoiding these high-risk behaviors further enhances adolescents’ mental health and lowers the risk of depression. Additionally, the self-determination theory (SDT) offers valuable insight into the relationship between meaning in life and depression. SDT (24) proposes that basic psychological needs form the basis for the development of self-motivation and personality integration. Only by meeting individuals’ basic psychological needs can there be sufficient motivation to promote individuals’ behavior and development, which is beneficial for mental health. Ryan and Deci (24) proposed three basic psychological needs: competence, relatedness, and autonomy. Hadden and Smith (25) further proposed that meaning in life is uniquely related to a set of well-being indicators. These indicators are closely associated with the three basic psychological needs proposed by Ryan and Deci (24), and can predict psychological well-being independently of the three psychological needs, meeting the criteria as basic psychological needs. Researchers have progressively begun to conceptualize the meaning in life as a basic psychological need and have validated its role in protecting and enhancing well-being and positive emotions (26). Steger et al. (12) proposed two relatively independent components of meaning in life: the presence of meaning in life (PML) and the search for meaning in life (SML). PML refers to individuals’ subjective perception of the meaning of life, and SML refers to individuals’ motivation and direction to search for meaning in life (12). The meaning-making model (27) distinguishes meaning from two aspects: global and situational. Global meaning involves the enduring beliefs and valued goals of individuals and directly affects their emotions and behaviors. As an internal cognitive resource, PML is part of global meaning (28). Situational meaning involves the evaluation and pursuit of the meaning of events, and its definition is similar to that of SML (27). Because situational meaning is associated with specific events, such as assessing specific events, and not to internal cognitive resources, SML may not directly affect emotions (28). Related studies have also shown that PML is more stably correlated with depression (28) and can negatively predict depression (9, 29). However, the relationship between SML and depression remains inconsistent across studies. For instance, some studies report a negative correlation between SML and depression (30), whereas others find a positive correlation (31), and some observe no correlation at all (28). Considerable controversy exists regarding the relationship between SML and depression (30–32). Therefore, this study only examined the relationship between PML and depression in adolescents and explored the underlying mechanisms. Based on previous research this study’s first hypothesis was that PML has a direct negative effect on depression among adolescents.

A sense of alienation refers to negative emotions, such as social isolation, oppression, and restraint, that arise from individuals becoming distant from their normal relationships with surrounding people, society, nature, and themselves and even being dominated or controlled (33). Studies have found that a sense of alienation negatively predicts meaning in life (34); however, there has been limited research on the impact of meaning in life on interpersonal connections or a sense of alienation. Olga and Maike (35) conducted a longitudinal study that revealed that meaning in life predicts belongingness and engagement in community activities and has an impact on the development of intimate relationships. Individuals with a higher sense of meaning in life are more likely to actively connect with others and the environment, leading to greater belongingness (35). According to SDT, when individuals’ basic psychological needs are not met, their intrinsic motivation to explore and learn are disrupted (24), and they may develop avoidance behaviors or lack active actions, such as reducing contact with others or society or feeling distant from their own behaviors (36), which may lead them to experience a sense of alienation from others, society, or the environment. Therefore, PML may affect the sense of alienation. Furthermore, adolescence is a sensitive period of perception and development (37), during which adolescents are likely to experience a strong sense of alienation (38). Alienation from friends, relatives, and the environment is not conducive to the establishment of good interpersonal relationships and interactions among adolescents and will also hinder their academic development, resulting in social adaptation difficulties (39, 40), thus leading to various crises. Studies have shown that the higher the sense of alienation among adolescents, the higher their level of depression (39). Therefore, this study’s second hypothesis was that sense of alienation mediates the relationship between PML and depression.

SDT focuses on internal growth trends and psychological needs, emphasizing the importance of satisfying basic psychological needs in constructing well-being (24). Life satisfaction, the perception and evaluation of quality of life according to individuals’ own standards, is a necessary component of well-being (41). A meta-analysis indicated a stable positive correlation between PML and life satisfaction (42) and that the PML could positively predict life satisfaction (9). Furthermore, positive well-being can prevent psychological disorders (43). Toshanloo (44) found that life satisfaction predicted the risk of developing depression over the subsequent three to six years. Lower levels of life satisfaction were associated with a higher risk of depression, indicating that reduced life satisfaction may be a potential risk factor for depression. Therefore, this study’s third hypothesis was that life satisfaction mediates the relationship between PML and depression.

Recent studies have shown that alienation from parents, peers, and oneself can lead to decreased life satisfaction among adolescents (45, 46). According to the supported main effect model (47), social resources are beneficial for improving well-being. When individuals perceive more connections and support, their life satisfaction further improves; when they lose their sense of connection and feel more alienated, their satisfaction with life decreases. Therefore, this study’s fourth hypothesis was that sense of alienation and life satisfaction sequentially mediate the relationship between PML and depression.

When exploring the relationships among PML, depression, sense of alienation, and life satisfaction among adolescents, gender should be given particular attention. First, there were gender-related differences in these variables. Studies have shown that adolescents of different gender experience different levels of depression (48), life satisfaction (49), and a sense of alienation (50). There is controversy regarding gender differences in the meaning in life. Studies have found that women report higher meaning in life than men (51), whereas others have not identified significant gender differences (52). Second, there may be significant gender differences in the relationships between the variables. According to the gender differentiation perspective, compared to men, women possess traits that lead to heightened emotional experiences when confronted with relational issues such as those involving interpersonal relationships (53). Therefore, women may experience greater stress than men when they feel alienated, leading to reduced well-being. This can result in decreased life satisfaction and exacerbate internalized emotional issues, such as depression. Meaning in life is a personal resource that helps adolescents deal with negative environments and distractions and maintain positive emotions (52, 54). As adolescence is a critical period for physical and cognitive development, there may be differences between genders in terms of life development, motivations, goals, and directions (51). Consequently, the impact of meaning in life on the emotions or well-being of male and female adolescents may differ (51). Currently, there is limited research on gender differences in the relationship between meaning in life and depression among adolescents, and findings are inconsistent. Yu et al. (55) found that PML was a better predictor of depressive symptoms in women than in men. However, other researchers have found that meaning in life is a strong predictor of depression in men (56). Therefore, this study further explored whether there were gender differences in the relationships among PML, depression, sense of alienation, and life satisfaction. Therefore, this study’s fifth hypothesis was that there would be gender differences in the chain mediation effects of sense of alienation and life satisfaction in the relationship between PML and depression.

**Figure 1:**
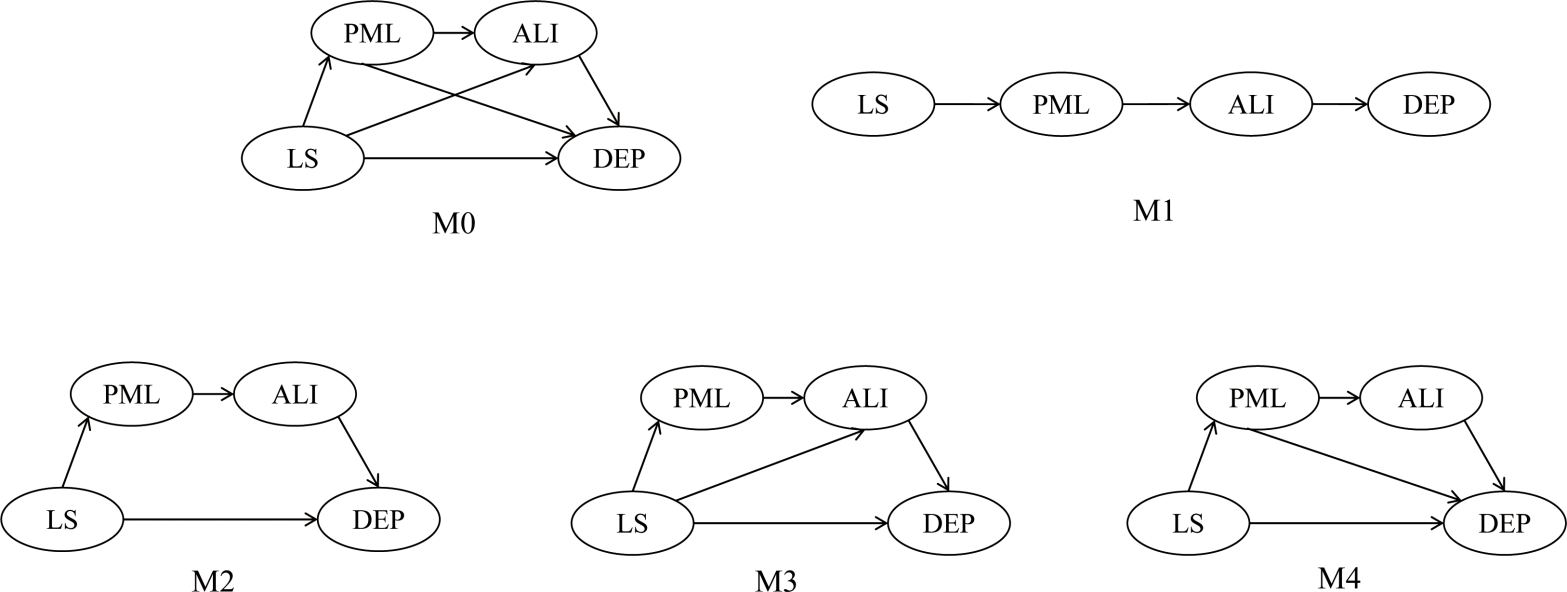
M0-M4. PML, Presenceof meaning in life; ALI, Sense of Alienation; LS, Life satisfaction; DEP, Depression.

In summary, based on SDT and the supported main effect model, a chain mediation model (**Figure 1**) was constructed to explore the relationship between PML and depression. Four hypotheses were proposed: PML would have a direct negative effect on depression (Hypothesis 1), a sense of alienation would mediate the relationship between PML and depression (Hypothesis 2), life satisfaction would mediate the relationship between PML and depression (Hypothesis 3), and a sense of alienation and life satisfaction would sequentially mediate the relationship between PML and depression (Hypothesis 4). Gender differences would be found in this chain mediation model (Hypothesis 5). We recruited third year students from a high school in Shandong, China, to participate in this study. The Chinese high school education system prioritizes core subjects and emphasizes academic achievement. The National College Entrance Examination (NCEE) is the primary pathway for Chinese high school students to enter universities, with results playing a pivotal role in admissions, leading to intense competition and high pressure. The China Youth & Children Research Center has collaborated with research institutions in Japan, South Korea, and the United States on a comparative study among high school students in these countries (57). The study found that Chinese high school students experience the highest academic pressure, focus intensely on academic achievement, and spend much of their extracurricular time on additional tutoring (57). Compared to their peers in other countries, Chinese high school students are more focused on academic performance and devote less attention to reflecting on their future or personal development (57). Chinese high school seniors who are under pressure to pursue higher education and major choices may show a higher level of confusion regarding the meaning and goals of life. Exploring this issue provides support and a basis for future mental health interventions for high school seniors.

## 2 Materials and methods

### 2.1 Participants

This study recruited senior students from a high school in Shandong, China, and the questionnaires were distributed offline. A total of 680 students volunteered to participate in our study, and through the screening, 621 participants (266 boys and 355 girls) were included, with a recovery rate of valid questionnaires being 91.32%. The ages ranged from 16-19, with a mean age of 17.09 ± 0.45 years. The exclusion criteria were as follows (1): Participants with highly consistent answers across all options were excluded. Reverse-scored items ensured variability, and consistent responses led to exclusion (2). Participants who failed attention-check items were excluded. These items required selecting specific options to assess participants’ engagement in the survey (3). Participants failing polygraph questions were excluded. These paired questions assessed the same concept using different wording and options. A discrepancy of over three points between paired responses indicated failure. This study included three pairs of polygraph questions. Participants were excluded if two or more pairs showed conflicting responses.

### 2.2 Measures

#### 2.2.1 Sense of alienation

The Adolescent students’ sense of alienation (ASAS) scale, developed by Yang et al. (33), measures the sense of alienation among Chinese adolescents. The scale comprises three dimensions: sense of social alienation (SAS; e.g., When facing troubles, I cannot rely on the collective or organization for moral support and assistance.), sense of interpersonal alienation (IAS; e.g., Even when I am with friends, I often feel lonely and isolated.), and sense of environmental alienation (EAS; e.g., I feel a sense of disconnection from nature.). Additionally, it includes three pairs of polygraph questions (6 items) designed to identify and exclude invalid responses. The scale contains a total of 52 items, including polygraph questions, with 10 reverse-scored items (e.g., “I feel close to the people around me”). A 7-point scale was used, ranging from 1 (not at all) to 7 (completely), with a higher average score indicating a stronger sense of alienation. The Cronbach’s α coefficients of social alienation, interpersonal alienation, and environmental alienation in this study were 0.94, 0.91, and 0.79, respectively. The Cronbach’s α coefficient for the overall ASAS was 0.96.

#### 2.2.2 Meaning in life

The Meaning in Life Questionnaire-Chinese version (MLQ-C) used in this study was revised by Wang (58). The questionnaire was originally developed by Steger et al. (12) and contains two dimensions: the presence of meaning (PML) and the search for meaning (SML). Liu and Gan (2010) adapted it to develop the Meaning in Life Questionnaire-Chinese version (MLQ-C), which has demonstrated good reliability and validity and has been widely used. The MLQ-C measures adolescents’ sense of meaning in life, was revised by Wang based on the adaptation of Liu and Gan (59). It consists of two dimensions: Presence of Meaning in Life (PML; e.g., “I understand the meaning of my life”) and Search for Meaning in Life (SML; e.g., “I am seeking the meaning of my life”) each containing five items. The PML dimension includes a reverse-scored item: “My life lacks a clear purpose”. The MLQ-C has demonstrated strong reliability and validity. The 10-item scale uses a 7-point scale ranging from 1 (not at all) to 7 (completely), with higher scores indicating a stronger sense of meaning in life. Only the PML dimension of the MLQ-C was used in this study, with a Cronbach’s α coefficient of 0.88.

#### 2.2.3 Life satisfaction

The Satisfaction with Life Scale (SWLS), originally developed by Diener et al. (60) and revised into Chinese version by Xiong and Xu (61) to measure life satisfaction of the general population in China, has been proven to have good reliability and validity. It has been widely used among Chinese adolescents and has demonstrated good reliability and validity (62). The scale consists of 5 items (e.g., I’m satisfied with my life) on a 7-point scale ranging from 1 (strongly disagree) to 7 (strongly agree), with higher scores indicating higher life satisfaction. The Cronbach’s α coefficient in this study was 0.80.

#### 2.2.4 Depression

The Beck Depression Inventory-II (BDI-II) is a depression self-rating scale developed by Beck et al. (63) and revised into Chinese version by Wang et al. (64). It has been widely used among Chinese adolescents and has demonstrated good reliability and validity (65). The scale consists of 21 items, rated on a 4-point scale ranging from 0 (not at all) to 3 (severely), e.g., “I do not feel sad” = 0, “I feel sad most of the time” = 1, “I feel sad all the time” = 2, “I am too sad or upset to bear” = 3. The total scores range from 0 to 63, with higher scores indicating higher depression severity. A score of 0-13 indicates minimal depression (no depression), mild depression scores range from 14-19, moderate depression scores range from 20-28, and severe depression scores range from 29-63. The Cronbach’s α coefficient in this study was 0.91.

### 2.3 Procedure

This study was approved by the Ethics Committee of the Institutional Review Board, and consent was obtained from both high school students and teachers. Parental/guardian consent was also obtained for minors. The experimenter, a graduate student professionally trained in psychology, was assisted by four high school teachers who were trained to distribute the questionnaires. The questionnaires were distributed across various classes, and each participant completed all four questionnaires in a single session. Participants were informed of the study’s purpose before completing the questionnaire, and informed consent was obtained from all participants. Questionnaires were completed anonymously to ensure participants’ privacy.

### 2.4 Statistical analysis

SPSS 26.0 was used for data organizing, descriptive statistics and correlation analysis. Amos24.0 was used to test the mediating effect and multigroup analysis.

## 3 Results

### 3.1 Common method bias

To avoid the potential common method bias, we conducted several control during the research procedure. For example, the questionnaires were completed anonymously, and certain items were reverse scored. Next, the original data were analyzed using the Harman single factor test. The results showed that 15 factors had eigenvalues greater than 1, with the first factor accounting for 29.51% of the variance, which was below the critical criterion of 40%, indicating that there was no serious common method bias in this study.

### 3.2 Descriptive statistics and correlation analysis

The correlations between the variables in this study are presented in **Table 1**. The results showed that PML (*M* = 4.40, *SD* = 1.48) was positively correlated with life satisfaction (*M* = 3.57, *SD* = 1.20; *r* = .40, *p* <.001), and negatively correlated with sense of alienation (*M* = 3.64, *SD* = 1.09; *r* = -.58, *p* <.001) and depression (*M* = 13.57, *SD* = 10.22; *r* = -.50, *p* <.001). Life satisfaction was negatively correlated with both sense of alienation (*r* = -.58, *p* <.001) and depression (*r* = -.53, *p* <.001). Sense of alienation was positively correlated with depression (*r* = .74, *p* <.001).

**Table 1:** Correlation analysis (N = 621).

Variables	1	2	3	4
1. PML	1			
2. ALI	-0.58^***^	1		
3. LS	0.40^***^	-0.58^***^	1	
4. DEP	-0.50^***^	0.74^***^	-0.53^***^	1

***p < 0.001. PML, Presence of meaning in life; ALI, Sense of Alienation; LS, Life satisfaction; DEP, Depression. Additionally, the data for all variables in the model were standardized.

### 3.3 Mediation analysis

#### 3.3.1 Item parcelling

According to **Table 1**, there was a significant correlation among PML, sense of alienation, life satisfaction, and depression. Thus, a structural equation model was constructed to test the mediation effect. To minimize random errors, improve modeling efficiency, and stabilize estimation, this study conducted item packaging processing. The factorial algorithm was used to package the BDI- II into three latent variables, while the internal consistency approach was used to package the three-dimensional ASAS scale into three latent variables (66).

#### 3.3.2 Mediation model testing

The direct effect of PML on depression was tested, showing a good model fit: χ^2^/df = 5.25, RMSEA = 0.08, SRMR = 0.07, CFI = 0.97, NFI = 0.97, TLI = 0.96. PML significantly and negatively predicted depression (γ = –.55, *p* <.001), supporting Hypothesis 1. Two mediator variables, sense of alienation and life satisfaction, were included in the structural equation model, and four competing models were constructed for evaluation. One model represented full mediation (M1), while the other three represented partial mediation models (M2~M4). As shown in **Table 2**, the data best fit the hypothesized model (M0) with the indices: χ2/df = 3.43, RMSEA = 0.06, SRMR = 0.05, CFI = 0.96, NFI = 0.95, TLI = 0.95. M0, proposed based on the study’s hypotheses, includes a full mediation model and multiple partial mediation models. The fit of the other three models (M1–M3) was inferior to that of the hypothesized model (M0). Although the fit of model M4 was similar to M0, we selected model M0 based on the principle of minimizing the Akaike Information Criterion (AIC) and the Bayesian Information Criterion (BIC), where smaller values indicate better model fit (67) (M0–M4, see **Figure 1**). The results of the chain mediation analysis indicated that PML significantly and negatively predicted sense of alienation (γ = –.66, *p* <.001), sense of alienation significantly and negatively predicted life satisfaction (γ = –.65, *p* <.001), and life satisfaction significantly and negatively predicted depression (γ = –.14, *p* <.001).

**Table 2:** Fit indices of the hypothesized model and the competing model.

Model	*χ^2^*/*df*	CFI	NFI	IFI	TLI	AIC	BIC	ECVI	RMSEA	GFI	AGFI
M0	3.43	0.96	0.95	0.96	0.95	412.52	580.91	0.67	0.06	0.94	0.91
M1	5.37	0.93	0.91	0.93	0.91	612.14	767.24	0.99	0.08	0.90	0.87
M2	4.98	0.93	0.92	0.93	0.92	569.63	729.16	0.92	0.08	0.91	0.88
M3	5.03	0.93	0.92	0.93	0.92	571.53	735.49	0.92	0.08	0.91	0.88
M4	3.43	0.96	0.95	0.96	0.95	413.39	587.35	0.67	0.06	0.94	0.91

The data of all variables in the model were standardized.

**Figure 2:**
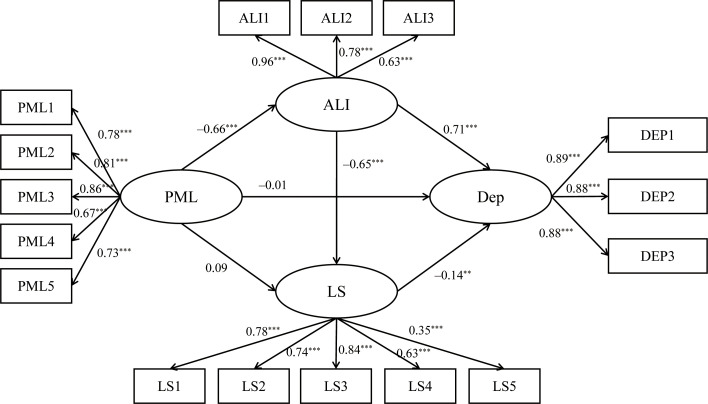
Chain mediation model. ***p<0.001, **p<0.01. PML, Presence of meaning in life; ALI, Sense of Alienation; LS, Life satisfaction; DEP, Depression.

The Bootstrap method was employed to test the mediating effect of sense of alienation and life satisfaction in the relationship between PML and depression. The number of iterations was 2000 and the confidence interval was 95%. The mediation effect was significant if the 95% confidence interval did not include 0 value. **Table 3** shows that the 95% confidence interval for the mediating effect of sense of alienation between PML and depression ranged from [–0.56, –0.39]. Since this confidence interval did not include zero, the mediating effect of sense of alienation was significant, with a mediating effect value of –0.47. This finding supports Hypothesis 2. The 95% confidence interval for the mediating effect of life satisfaction between PML and depression was [–0.04, 0.00]. As this confidence interval included zero, the mediating effect was not significant, and Hypothesis 3 was not supported. The 95% confidence interval for the serial mediating effect of alienation and life satisfaction between PML and depression was [–0.10, –0.02]. As this confidence interval did not include zero, the serial mediating effect was significant, with a mediating effect value of –0.06. This finding supports Hypothesis 4. Consequently, sense of alienation and life satisfaction acted as sequential mediators in the relationship between PML and depression among high school students (**Figure 2**).

**Table 3:** Bootstrap analysis of mediation effect.

Mediating path	Indirect effect	Effect size	95%CI	
			Lower	Upper
PML→DEP	–0.006	1.09%	–0.088	0.078
PML→ALI→DEP	–0.471	85.79%	–0.562	–0.393
PML→LS→DEP	–0.012	2.19%	–0.039	0.002
PML→ALI→LS→DEP	–0.060	10.93%	–0.101	–0.022
Total indirect effect	–0.543	98.91%	–0.613	–0.480
Total effect	–0.549		–0.614	–0.477

PML, Presence of meaning in life; ALI, Sense of Alienation; LS, Life satisfaction; DEP, Depression. The data of all variables in the model were standardized.

### 3.4 Moderating effect analysis of gender

To explore whether gender moderates the chain mediation effect, a multiple group analysis was employed to test the gender differences. Mediation effect models were firstly tested separately for boys (M5) and girls (M6), both demonstrating satisfactory fit indices. This confirmed the appropriateness of performing a multiple-group comparison analysis. Subsequently, the freely estimated model (M7) and the constrained model (M8) were separately constructed. Since the M7 model was nested within the M8 model, a nested model comparison was performed (68). The results indicated a significant difference between the two competing models (Δχ^2^ = 37.16, df = 18, *p* < 0.01), suggesting that there was a significant gender difference in the chain mediation effect (see **Table 4**).

**Table 4:** Fit indices of the cross-group structural equation model.

Model	χ2/df	CFI	NFI	IFI	TLI	RMSEA
M5	2.39	0.94	0.91	0.94	0.93	0.07
M6	2.30	0.97	0.94	0.97	0.96	0.06
M7	2.34	0.96	0.93	0.96	0.95	0.05
M8	2.32	0.96	0.92	0.96	0.95	0.05

The data of all variables in the model were standardized.

**Figure 3:**
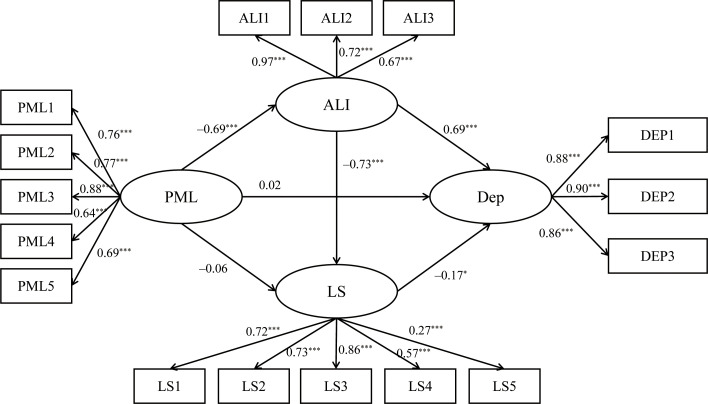
Chain mediation model for males. ***p<0.001, *p<0.05. PML, Presenceof meaning in life; ALI, Sense of Alienation; LS, Life satisfaction; DEP, Depression.

**Figure 4:**
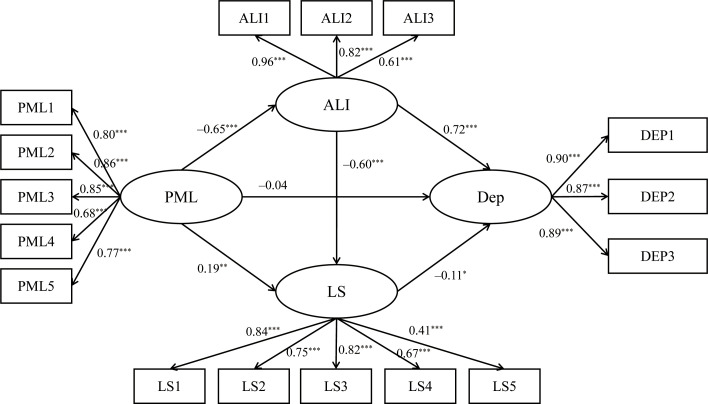
Chain mediation model for females. ***p<0.001, **p<0.01, *p<0.05. PML, Presenceof meaning in life; ALI, Sense of Alienation; LS, Life satisfaction; DEP, Depression.

Specifically, in the model for boys (see **Figure 3**), PML influenced depression through two pathways. First, PML negatively predicted sense of alienation (*γ* = –.69, *p* < 0.001), which in turn positively predicted depression (*γ* = .69, *p* < 0.001). The 95% confidence interval for the mediating effect was [–0.62, –0.34], indicating a significant indirect effect of PML on depression via sense of alienation. Second, sense of alienation significantly negatively predicted life satisfaction (*γ* = –.73, *p* < 0.001), and life satisfaction significantly negatively predicted depression (*γ* = –.17, *p* < 0.001). The 95% confidence interval for the chain mediation effect was [–0.17, –0.02], indicating that PML indirectly affected depression through both sense of alienation and life satisfaction. In the model for girls (see **Figure 4**), PML influenced depression through three pathways. First, PML negatively predicted sense of alienation (*γ* = –.65, *p* < 0.001), which in turn positively predicted depression (*γ* = .72, *p* < 0.001). The 95% confidence interval for the mediating effect was [–0.59, –0.38], indicating that PML indirectly affected depression through sense of alienation. Second, PML positively predicted life satisfaction (*γ* = .19, *p* < 0.01), and life satisfaction negatively predicted depression (*γ* = –.11, *p* < 0.05), the 95% confidence interval for the mediating effect was [–0.06, –0.01], indicating that PML indirectly affected depression through life satisfaction. Finally, PML negatively predicted sense of alienation (*γ* = –.65, *p* < 0.001), which negatively predicted life satisfaction (*γ* = –.60, *p* < 0.001), and life satisfaction negatively predicted depression (*γ* = –.11, *p* < 0.05). The 95% confidence interval for the chain mediation effect was [–0.09, –0.01], indicating that PML indirectly affected depression through both sense of alienation and life satisfaction.

## 4 Discussion

This study established a chain mediation model to investigate the relationship between PML and depression among Chinese high school seniors. The results revealed the direct negative effects of PML on depression (Hypothesis 1), mediating role of a sense of alienation (Hypothesis 2), and chain mediating role of a sense of alienation and life satisfaction (Hypothesis 4). However, the study did not find the mediating effect of life satisfaction between PML and depression (Hypothesis 3 was not supported). Furthermore, this study revealed gender differences in the model (Hypothesis 5).

First, we found a significant negative correlation between PML and depression and that PML had a significant direct negative effect on depression, which was consistent with the findings of previous studies. Relevant studies have shown a stable correlation between PML and depression (28, 69). Furthermore, other researchers have found that PML negatively predicts depressive symptoms (9, 29). Our study suggests that PML is a potential factor affecting the mental health of adolescents who are striving to establish a sense of identity. Having a sense of meaning is the goal of life and a means to enrich it (7). Adolescents who perceive higher meaning in life are more likely to experience self-worth (70), develop a sense of self-identity (7, 71), and experience fewer negative emotions.

Second, the impact of PML on depression can be mediated by a sense of alienation. Lower PML leads to a higher sense of alienation, which in turn leads to higher levels of depression. According to the SDT (24), basic psychological needs help promote the internalization and integration of intrinsic and extrinsic motivation in behavior and are key factors in developing and maintaining motivation. If a behavior or task is attractive to an individual, basic psychological needs can maintain this intrinsic motivation, thereby promoting contact and exploratory behaviors. However, if a behavior or task lacks intrinsic attraction to an individual, basic psychological needs must be internalized and integrated with external motivation to promote the behavior (24). As a perception of the meaning of life (12), PML is an important psychological need for adolescents in self-development and improvement. When PML cannot be well satisfied, the internalization and integration of adolescents’ behavioral motivation will have problems, resulting in some isolation and avoidance behaviors. In addition, low PML makes adolescents have negative and critical views of themselves (72), all of which lead to a high sense of alienation. A sense of alienation is associated with mental health problems (73). Compared with adolescents with lower levels of sense of alienation, those with higher levels of sense of alienation experience less belonging and higher levels of stress and even develop problematic behaviors (74, 75), which can trigger mental health problems, such as depression.

This study did not identify a mediating role of life satisfaction between PML and depression. While this result seems to contradict previous understanding, the cultural context may help explain it. The participants in this study were Chinese adolescents, who come from a predominantly collectivist cultural background. Steger et al. (76) proposed the dialectic model of meaning in life to discuss cultural differences in how life’s meaning is perceived. They argued that individuals in individualistic cultures prioritize the presence of meaning, using the perception and understanding of life’s meaning as strategies for self-improvement. Conversely, actively seeking meaning may undermine self-image (31, 76). In contrast to those in individualistic cultures who focus on achieving successful outcomes to build a positive self-perception, people in collectivistic cultures place greater emphasis on the process itself, which involves both effort and self-improvement as well as exploration and the search for meaning (Steger et al., 2008). Thus, while individualism emphasizes the presence of meaning in life, collectivism may prioritize the search for meaning over its presence. Within the Chinese cultural context, actively searching for meaning may promote an individual’s sense of well-being. Additionally, our study sample comprised Chinese high school seniors preparing for the National College Entrance Examination. In China, this examination is considered one of the most significant, often viewed as a determinant of an individual’s future. Consequently, parents and teachers impose high expectations and strict demands on students, resulting in elevated academic and life pressures. Such pressures may further moderate the relationship between PML and life satisfaction, with excessive pressure potentially reducing adolescents’ life satisfaction. This finding highlights the importance of further exploring how different dimensions of meaning in life affect well-being across diverse cultural contexts.

Third, this study found that the effect of PML on depression could be mediated by the chain mediation of the sense of alienation and life satisfaction. The sense of alienation negatively affected life satisfaction, which was consistent with the results of previous studies (45). High life satisfaction is inseparable from the support of social resources (46); the higher the sense of alienation, the lesser social support is perceived, which is not conducive to improving life satisfaction. The results of this study indicate that for high school senior students, a lower level of PML leads to a higher sense of alienation, which further reduces their well-being, leading to a decrease in life satisfaction and an increase in depression. Depression and life satisfaction are important dimensions of well-being. The current results are consistent with those of the SDT (24), supporting the role of basic psychological needs in well-being. Additionally, Stillman et al. (77) proposed that meaning in life promotes interpersonal relationships. Further studies found that those who self-reported a higher meaning in life were assessed by others as having higher interpersonal attractiveness (78). Stillman et al. (78) believed that individuals with strong meaning in life were ideal social interactors, and individuals tended to avoid those without purpose and meaning in life. This further explains why individuals with a high PML tend to have a lower sense of alienation and higher well-being. This is because they are generally less socially isolated and more satisfied with their own state and life, which helps prevent depression.

Finally, this study found significant gender differences in the mediating effect of life satisfaction on the relationship between PML and depression. In women, life satisfaction mediates the relationship between PML and depression, whereas in men, the mediating effect is not significant, primarily because PML does not predict life satisfaction. From the perspective of emotional arousal and perception, when the PML is satisfied as a basic psychological need, individuals tend to perceive stronger positive emotions (50), leading to greater satisfaction with their current lives. As children progress through adolescence, their emotional intensity increases, and their perception of emotions becomes more sensitive (79). From the perspective of gender socialization, girls are encouraged from a young age to fully experience their emotions, whereas boys are taught to be strong and strive to control and suppress them (80). Therefore, compared to adolescent boys, adolescent girls tend to have a more sensitive and broader perception of emotions (50), and positive emotions elicited by a high PML are more readily captured by adolescent girls. As a result, PML had a more significant positive effect on the life satisfaction of adolescent girls than on the life satisfaction of adolescent boys. In addition, from the perspective of psychological resources, PML, as a positive internal psychological resource, contributes to the maintenance and promotion of positive emotions and mental health (81, 82). Our findings are similar to those of Song et al. (83), who found that psychological resources have a significant buffering effect on depression levels in adolescent girls than in adolescent boys. Our results suggest that compared to adolescent boys, adolescent girls tend to be more adept at harnessing PML, a valuable internal psychological resource, to uphold their own positive emotions.

This study deepens our understanding of the relationship among adolescents’ meaning in life, sense of alienation, life satisfaction, and depression, providing valuable insights for fostering healthy development in adolescents. First, both schools and families should acknowledge the essential role of meaning in life in adolescents’ psychological development and overall well-being. They should not focus solely on academic performance but also support adolescents in exploring and finding meaning in life, ensuring the fulfillment of their basic psychological needs, and encouraging the active pursuit of life’s purpose to prevent psychological problems. Secondly, meaning in life has no significant positive effect on boys’ life satisfaction compared to girls. Therefore, both school and family education should focus more on helping boys increase their awareness and application of basic psychological needs.

This study had some limitations. First, this was a cross-sectional study and could not reveal a causal relationship between PML and depression. Second, this study only included the PML dimension of meaning in life in the analysis model, without incorporating the SML dimension. However, other researchers have identified an interaction between PML and SML, with PML predicting SML one year later (84). One possible reason for this is that individuals may search for meaning because their current perception of meaning is insufficient. Therefore, future research could include both the PML and SML in the model and conduct longitudinal studies to clarify the relationship between meaning in life and depression, as well as the relationship between the two dimensions of meaning in life. Third, this study did not consider participants’ social class or the socio-economic status of their families; however, these factors may influence the variables explored in the current study. Marquez and Long (85) have investigated the impact of socio-economic status on life satisfaction among adolescents from 46 countries. Their study found that students from higher socio-economic backgrounds reported greater life satisfaction compared to those from lower socio-economic backgrounds. However, the decline in life satisfaction was more pronounced among those from higher socio-economic backgrounds. Therefore, socio-economic status and other factors may influence the underlying mechanisms explored in the present study, and future research should further investigate their effects. Finally, only high school seniors were selected as participants. The third year of Chinese high school is a crucial stage for high school students who face preliminary planning for future majors and life goals, which is special and typical. Therefore, generalizing the results to adolescents of other age groups requires caution.

In general, this study reveals the potential pathways through which PML affects depression among high school seniors in the Chinese context and verifies the important role of PML as a basic psychological need in the psychological health development of this group. This study expands and supplements research on meaning in life among adolescents and provides a reference for improving the mental health of adolescents.

## Data Availability

The raw data supporting the conclusions of this article will be made available by the authors, without undue reservation.
